# Analysis of striatal connectivity corresponding to striosomes and matrix in de novo Parkinson’s disease and isolated REM behavior disorder

**DOI:** 10.1038/s41531-024-00736-9

**Published:** 2024-06-25

**Authors:** S. Marecek, T. Krajca, R. Krupicka, P. Sojka, J. Nepozitek, Z. Varga, C. Mala, J. Keller, J. L. Waugh, D. Zogala, J. Trnka, K. Sonka, E. Ruzicka, P. Dusek

**Affiliations:** 1 Department of Neurology and Center of Clinical Neuroscience, First Faculty of Medicine, Charles University and General University Hospital in Prague, Prague, Czech Republic; 2 Czech Technical University in Prague, Faculty of Biomedical Engineering, Kladno, Czech Republic; 3 Department of Radiodiagnostics, Na Homolce Hospital, Prague, Czech Republic; 4 Division of Pediatric Neurology, Department of Pediatrics, University of Texas Southwestern, Dallas, TX USA; 5 Institute of Nuclear Medicine, First Faculty of Medicine, Charles University and General University Hospital, Prague, Czech Republic

**Keywords:** Parkinson's disease, Neurodegeneration, Diagnostic markers, Neurodegeneration

## Abstract

Striosomes and matrix are two compartments that comprise the striatum, each having its own distinct immunohistochemical properties, function, and connectivity. It is currently not clear whether prodromal or early manifest Parkinson’s disease (PD) is associated with any striatal matrix or striosomal abnormality. Recently, a method of striatal parcellation using probabilistic tractography has been described and validated, using the distinct connectivity of these two compartments to identify voxels with striosome- and matrix-like connectivity. The goal of this study was to use this approach in tandem with DAT-SPECT, a method used to quantify the level of nigrostriatal denervation, to analyze the striatum in populations of de novo diagnosed, treatment-naïve patients with PD, isolated REM behavioral disorder (iRBD) patients, and healthy controls. We discovered a shift in striatal connectivity, which showed correlation with nigrostriatal denervation. Patients with PD exhibited a significantly higher matrix-like volume and associated connectivity than healthy controls and higher matrix-associated connectivity than iRBD patients. In contrast, the side with less pronounced nigrostriatal denervation in PD and iRBD patients showed a decrease in striosome-like volume and associated connectivity indices. These findings could point to a compensatory neuroplastic mechanism in the context of nigrostriatal denervation and open a new avenue in the investigation of the pathophysiology of Parkinson’s disease.

## Introduction

Parkinson’s disease (PD) is a common neurodegenerative disorder associated with abnormal aggregation of α-synuclein in neurons. Its hallmark motor manifestation, bradykinesia in combination with resting tremor and/or rigidity, is caused in large part by degeneration of the substantia nigra pars compacta (SNc)^1,2^. The degeneration of SNc starts predominantly at the ventrolateral part, with loss of striatal dopamine innervation being nearly complete in the putamen with a relative sparing of the caudate, except its most dorsal rostral part^3–5^. PD may have a prodromal period manifested by various non-motor symptoms^6^. Isolated rapid eye movement sleep behavior disorder (iRBD) is a highly specific marker of this prodromal stage in PD, though iRBD can also be a prodromal feature of other synucleinopathies, such as dementia with Lewy bodies and multiple system atrophy. In general, RBD is a parasomnia characterized by loss of normal muscle atonia and dream enactment behavior in rapid eye movement (REM) sleep. Isolated RBD is caused by neurodegeneration of the nucleus subcoeruleus and associated brainstem nuclei responsible for maintaining muscle atonia in REM sleep. Thus, iRBD presents a promising condition for exploring potential preventive and therapeutic interventions for synucleinopathies^7–9^.

The striatum, the primary input site of the basal ganglia, plays a crucial part in the pathophysiology of PD symptoms^10^. The main cell population of the human striatum consists of GABAergic medium spiny neurons (MSNs), which can be further divided into subpopulations based on several criteria: MSNs of the direct and indirect pathways, neurons of ventromedial and dorsolateral striatal regions, and of striosomes and matrix^11,12^. Striosomes and matrix can be identified based on immunohistochemical properties with differential expression of more than 60 proteins^13,14^. The matrix-to-striosome ratio in the striatum is approximately 85:15. Although there is some overlap, the spatial distribution and structural connectivity (afferent and efferent) of striosomes and matrix are distinct in several ways. Striosomes are found throughout the whole striatum, but predominantly in the rostral, ventral, and medial parts^15^. Striosomal afferents are mainly from limbic-associated cortical and subcortical regions while matrix afferents have mainly somatosensory and motor cortical origins^16–18^. Most striosomal MSNs appear to project into the nuclei of the direct pathway, whereas the matrix MSNs are evenly divided between direct and indirect pathway targets^19–21^. Both striosomes and matrix receive innervation from neurons in the SN, with striosomes primarily connected to neurons in the ventral tier of SNc and the densocellular zone of the substantia nigra pars reticulata (SNr), while the matrix primarily receives afferents from the dorsal tiers of SNc and the ventral tegmental area^22–24^. Notably, only striosomal MSNs project to dopamine-containing neurons of SNc^11,13,19^. Furthermore, animal studies on rats and mice discovered that striosomes play a role in learning new behaviors^25,26^.

Several diseases and disorders disproportionally affect striosomes or matrix, including Huntington’s disease, dopamine-responsive dystonia, and drug addiction^13^. In earlier stages of multiple system atrophy, a preferential degeneration of matrix MSNs has been found, although in later stages, the degeneration affects both striosomes and matrix equally^27,28^. The number of studies on pathologies of striosomes and matrix in PD is limited. Although no differences were found in striosomal and striatal matrix volumes in a histopathological study in patients with PD^27^, functional alteration of matrix and striosomes can be expected in early PD stages since they are innervated by distinct areas of SNc with different degrees of degeneration^5,11,13,29,30^. There are also hints of over-activation of striosomes in levodopa induced dyskinesias in PD^31^. To our knowledge, there have been no studies on the role of striosomes and matrix in iRBD.

Although striosomes and matrix have distinct distribution, function, and connectivity, their identification through imaging has not been possible until recently. Based on differential connectivity, demonstrated through tract tracing studies in animals, Waugh et al. performed a series of probabilistic striatal parcellations with the goal to identify voxels with striosome-like and matrix-like patterns of connectivity. The “-like” suffix is based on two of the limitations of the method: 1) in human tissue, striosomes are islands of neurons with a maximum diameter of ∼1.25 mm. Thus, with the typical spatial resolution of diffusion MRI (2 mm isotropic) striosome-like voxels inevitably have some contribution of matrix tissue and 2) the method identifies voxels with a connectivity pattern of either striosomes or matrix, which should not be conflated with actually describing neurons using immunohistochemical staining^32^.

The aim of this study is to assess possible changes in striosome- and striatal matrix-associated connectivity in the context of nigrostriatal denervation in patients with PD, iRBD, and controls.

## Results

### Subject characteristics

Our study cohort consisted of 72 patients with PD, 56 patients with iRBD and 45 healthy controls (Table 1). iRBD group had a higher proportion of men, was older, and had lower MoCA scores compared to controls. PD group had higher MDS-UPDRS III score compared to iRBD and control groups. We did not find significant inter-group differences in the cortical thickness of the striosome- and matrix-favoring composite masks, nor in the total volumes of striatal masks, putamina, and caudates.

**Table 1:** Demographic and clinical characteristics, and other possible confounders

Characteristics	Controls (*n* = 45)	iRBD (*n* = 56)	PD (*n* = 72)	Inter-group *p*-value	Post-hoc tests
Male sex (%)	30 (67)	48 (86)	47 (45)	**0.023**	iRBD>PD, C
Age (years)	60.4 ± 9.1	66.8 ± 6.6	60.0 ± 12.1	**<0.001**	iRBD>>>PD, C
MoCA	25.6 ± 2.4	23.6 ± 2.9	24.8 ± 3.0	**0.028**^**a**^	C>iRBD
MDS-UPDRS III	3.3 ± 3.9	6.6 ± 5.7	31.0 ± 13.3	**<0.001**^**a**^	PD>>>iRBD, C
MDS-UPDRS III - tremor	0.9 ± 1.3	2.1 ± 2.4	6.3 ± 4.1	**<0.001**^**a**^	PD>>>iRBD, C
MDS-UPDRS III - bradykinesia	1.9 ± 2.1	3.5 ± 3.8	18.2 ± 8.4	**<0.001**^**a**^	PD>>>iRBD, C
MDS-UPDRS III - rigidity	0.3 ± 1.1	0.2 ± 0.6	4.1 ± 2.9	**<0.001**^**a**^	PD>>>iRBD, C
MDS-UPDRS III - axial	0.2 ± 0.6	1.0 ± 1.0	2.5 ± 1.9	**<0.001**^**a**^	PD>>>iRBD, C
Disease duration^b^	n.d.	7.0 ± 5.2	2.1 ± 1.8	n.d.	n.d.
Total striatal seed mask volume (cm^3^)	14.3 ± 1.7	14.1 ± 1.7	14.8 ± 1.7	0.883^c^	–
Putaminal volume (cm^3^)	8.6 ± 1.1	7.9 ± 1.3	8.4 ± 1.6	0.394^c^	–
Caudate volume (cm^3^)	6.8 ± 0.9	6.7 ± 0.9	6.9 ± 0.9	0.663^c^	–
Striosome-favoring target mask volume (cm^3^)	16.0 ± 1.9	15.9 ± 1.7	16.3 ± 1.8	0.700^c^	–
Matrix-favoring target mask volume (cm^3^)	77.2 ± 9.8	80.5 ± 11.3	79.4 ± 10.4	0.883^c^	–
Cortical thickness of matrix-favoring cortical areas (mm)	2.13 ± 0.10	2.12 ± 0.10	2.14 ± 0.12	0.855^a^	–
Cortical thickness of striosome-favoring cortical areas (mm)	2.90 ± 0.10	2.88 ± 0.12	2.88 ± 0.12	0.623^a^	–

Where applicable, the data are presented as mean ± standard deviation. *MoCA* Montreal Cognitive Assessment, *MDS-UPDRS* Movement Disorder Society-Sponsored Revision of the Unified Parkinson’s Disease Rating Scale, *C* controls, > for *p* < 0.05, >>> for *p* ≤ 0.001.

^a^Adjusted for age and sex.

^b^Disease duration is defined as subjective duration of dream enactment behavior in iRBD and time since the occurrence of first motor symptom in PD.

^c^Adjusted for age, sex, and total intracranial volume.

*p* < 0.05 values are highlighted in bold.

### Compartmental volume

**Fig. 1:**
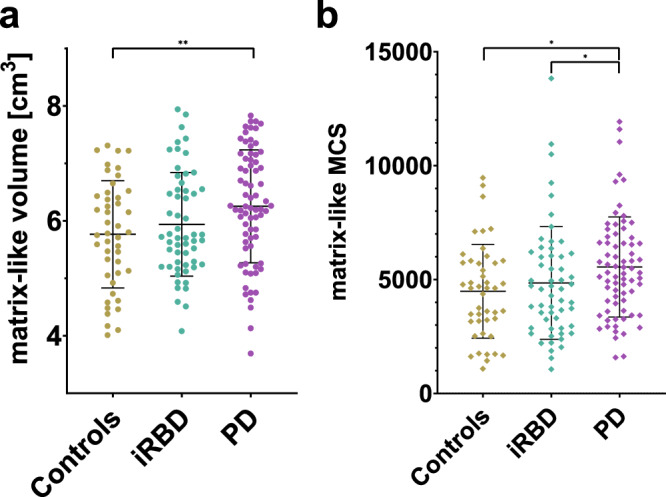
Differences in matrix-like volume and associated MCS between groups. Scatter plot with values of matrix-like compartmental volume (**a**) and mean connectivity scores of both hemispheres to matrix-favoring brain structures (**b**) in PD, iRBD, and controls. Volumes adjusted to the mean total striatal mask volume. The matrix-like voxels were selected based on their connectivity bias of at least 0.87 to matrix-favoring target structures. Line and whiskers represent mean and standard deviations. *<0.05, **<0.01; *MCS* mean connectivity score.

We found an inter-group difference in matrix-like compartmental volume [*F*(2,168) = 3.701, *p* = 0.027, partial *η*^2^ = 0.042], with PD patients having a higher number of matrix-like voxels than controls (*p* = 0.008) in the post-hoc analysis (Fig. 1a). The differences in striosome-like compartmental volume were not significant (Table 2).

**Table 2:** Comparison of compartmental volume, iSD, MCS in PD, iRBD, and controls

	Controls (*n* = 45)	iRBD (*n* = 56)	PD (*n* = 72)	Inter-group *p*-value	partial *η*^2^	Between groups
Matrix-like volume [cm^3^]	5.8 ± 0.9	5.9 ± 0.9	6.3 ± 1.0	**0.027**	0.042	PD>>C
Matrix-like MCS	4485 ± 2056	4852 ± 2474	5552 ± 2199	**0.024**	0.043	PD>iRBD, C
Matrix-like iSD [%]	50.7 ± 7.3	49.9 ± 7.0	52.9 ± 8.1	0.162	–	–
Striosome-like volume [cm^3^]	2.4 ± 0.9	2.0 ± 0.8	2.3 ± 0.9	0.174	–	–
Striosome-like MCS	404 ± 348	317 ± 303	355 ± 375	0.341	–	–
Striosome-like iSD [%]	33.9 ± 11.2	29.0 ± 10	31.9 ± 11.2	0.288	–	–

Where applicable, the results are shown as mean ± standard deviation, with volumetric values adjusted to the mean total striatal mask volume. *MCS* mean connectivity score, *iSD* index of streamline density, *C* controls, > for *p* < 0.05, >> for *p* ≤ 0.01

*p* values <0.05 highlighted in bold.

As the iRBD subjects were significantly older than the PD subjects, we performed a sensitivity analysis in age-matched subgroups, yielding 50 PD subjects (mean age 66.3 ± 8.3) and 50 iRBD subjects (mean age 66.6 ± 7.0). Using these subject groups, we found no significant differences in compartmental volumes between PD and iRBD subject groups. 

In the control group, voxels identified as tiresome-like constituted 15.4% of the total striatal volume. Voxels exceeding the defined threshold of 0.87 were found to be 2.8 times more likely to be matrix-like than striosome-like. These results are roughly in line with the results of Waugh et al.^32^, where striosome-like voxels made up 15.9% of striatal volume, and supra-threshold voxels were 2.2 times more likely to be in the matrix-like compartment.

The change to a stricter 95% compartment identification threshold did not negatively affect the results of analysis of compartmental volumes, their associations with nigrostriatal denervation, nor correlations with MDS-UPDRS III and its motor subscores (Supplementary Tables 1–3).

### Mean connectivity score and index of streamline density

We observed an inter-group difference in the MCS to matrix-favoring target regions [*F*(2,168) = 3.798, *p* = 0.024, partial *η*^2^ = 0.043] (Table 2). In the post-hoc analysis, the MCS was significantly higher in PD patients than in controls (*p* = 0.013) and in iRBD patients (*p* = 0.045) (Fig. 1b). We did not find significant inter-group differences in MCS to striosome-favoring regions, nor in the matrix- and striosome-like iSD.

As with the previous analysis of compartmental volumes we performed a sensitivity analysis in age-matched subgroups, yielding 50 PD subjects (mean age 66.3 ± 8.3) and 50 iRBD subjects (mean age 66.6 ± 7.0). Using these subject groups, the inter-group difference in the MCS to matrix-favoring target regions remained significant [*F*(1,96) = 4.677, *p* = 0.033, partial *η*^2^ = 0.046].

### Inter-subject effects of nigrostriatal denervation

**Fig. 2:**
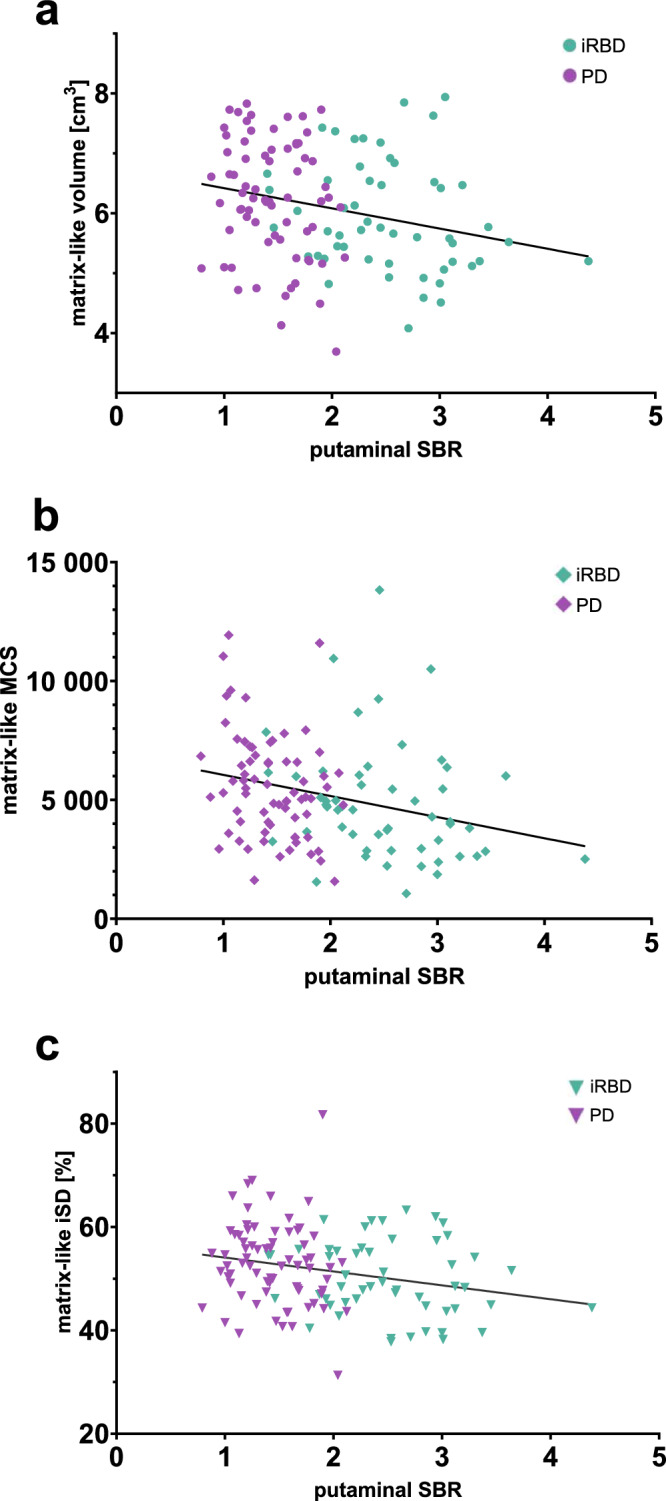
Putaminal SBR in relation to matrix-like volume and matrix-associated connectivity indices. Scatter plots representing the relation of SBR with mean matrix-like compartmental volume (**a**), mean connectivity score (**b**), and streamline density index (**c**) of both hemispheres. The values of the matrix-like compartmental volume are adjusted to the mean total striatal mask volume. PD and iRBD data are pooled. *MCS* mean connectivity score; *iSD* streamline density index.

Controlling for age and sex, a negative correlation was observed between the mean putaminal SBR and the matrix-like compartmental volume, MCS, and iSD to matrix-favoring regions in a merged pool of PD and iRBD patients (Table 3) (Fig. 2). No significant correlation was found between striosome-like compartmental parameters and nigrostriatal denervation.

**Table 3:** Associations of nigrostriatal denervation (SBR) and compartmental volumes, iSD, and MCS

	Partial correlation coefficient	*p*-value
Matrix-like volume	−0.226	**0.012**
Matrix-like MCS	−0.265	**0.003**
Matrix-like iSD	−0.202	**0.025**
Striosome-like volume	−0.036	0.691
Striosome-like MCS	−0.007	0.936
Striosome-like iSD	−0.035	0.701

Using two-tailed partial correlation, *n* = 125. Controlling for age and sex (compartmental volumes, iSDs, and MCSs).

*MCS* mean connectivity score, *iSD* index of streamline density.

*p* < 0.05 values highlighted in bold.

### Intra-subject effects of nigrostriatal denervation

**Fig. 3:**
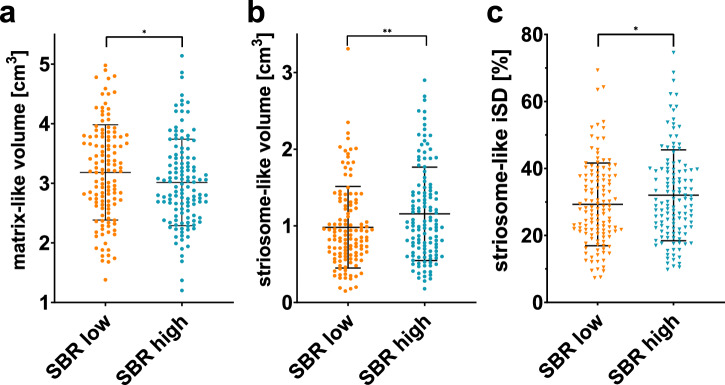
Putaminal SBR-based side differences in compartmental volumes and striosome-like iSD. Scatter plots values of matrix-like (**a**) and striosome-like (**b**) compartmental volumes and streamline density index to striosome-favoring brain regions (**c**). PD and iRBD data are pooled. Line and whiskers represent mean and standard deviations. *<0.05; **<0.01. iSD streamline density index.

Paired Student’s t-test between the side with lower and the side with higher SBR in pooled data from PD and iRBD groups showed significant differences in the matrix-like compartmental volumes, the striosome-like compartmental volumes, and the iSDs associated with striosome-favoring targets (Table 4) (Fig. 3). The average difference of putaminal SBR between the sides was 13.8%.

**Table 4:** Compartmental volume, iSD, MCS, and SBR side comparison based on nigrostriatal denervation

	Low SBR	High SBR	*p*-value	Cohen’s *d*
Putaminal SBR	1.77 ± 0.74	2.03 ± 0.69	**<0.001**	−1.26
Matrix-like volume	3.18 ± 0.80	3.01 ± 0.73	**0.036**	0.19
Matrix-like MCS	5319 ± 2733	5185 ± 2662	0.574	–
Matrix-like iSD	52.1 ± 9.0	51.1 ± 9.1	0.2	–
Striosome-like volume	0.98 ± 0.53	1.16 ± 0.61	**0.004**	−0.26
Striosome-like MCS	304 ± 387	389 ± 507	0.094	–
Striosome-like iSD	29.3 ± 12.3	32.0 ± 13.6	**0.042**	−0.18

Using two-tailed paired samples *t*-test, *n* = 125. The results are shown as mean ± standard deviation, with volume values adjusted to the total striatal mask volume. Each item was tested as the side with higher putaminal specific binding ratio versus the side with lower putaminal specific binding ratio. Volumes are measured in cm^3^, iSDs in %, and MCSs are measured as a connectivity score.

*MCS* mean connectivity score, *iSD* index of streamline density, *SBR* specific binding ratio.

*p* < 0.05 values are highlighted in bold.

### Associations with motor symptoms severity

Controlling for age and sex, a positive correlation was shown only between the tremor score and the iSD to matrix-favoring regions (*p* = 0.027, partial correlation coefficient = 0.261). However, using Paired Student’s *t*-test we found several significant differences between the sides with lower and the sides with higher MDS-UPDRS III scores and subscores (Table 5). Subjects with identical scores on both sides were excluded, yielding 71 subjects for MDS-UPDRS III score, 61 subjects for tremor subscore, 57 subjects for rigidity subscore, and 64 subjects for bradykinesia subscore.

**Table 5:** Compartmental volume, iSD, and MCS side comparison based on lateralized MDS-UPDRS III and its subscores in PD

	MDS-UPDRS III		Tremor		Rigidity		Bradykinesia	
	Low	High	Low	High	Low	High	Low	High
Matrix-like volume	**3.0** ± 0.6*	**3.3** ± **0.7**	3.0 ± 0.6	3.2 ± 0.7	**3.0** ± 0.6**	**3.3** ± **0.7**	3.0 ± 0.6	3.2 ± 0.7
Matrix-like MCS	5384 ± 2803	5741 ± 2368	5411 ± 2721	5676 ± 2491	5213 ± 2653	5546 ± 2469	5387 ± 2796	5633 ± 2444
Matrix-like iSD	52.6 ± 9.4	53.3 ± 9.0	52.1 ± 9.5	52.6 ± 8.7	52.9 ± 10.1	54.1 ± 9.2	52.6 ± 9.5	52.6 ± 9.2
Striosome-like volume	**1.2** ± 0.6*	**1.0** ± **0.6**	1.2 ± 0.6	1.1 ± 0.6	**1.3** ± 0.6*	**1.0** ± **0.6**	1.2 ± 0.7	1.0 ± 0.6
Striosome-like MCS	**434** ± 542*	**284** ± **361**	379 ± 515	294 ± 380	**442** ± 566*	**281** ± **392**	426 ± 569	295 ± 374
Striosome-like iSD	33.7 ± 14.0	30.3 ± 13.3	32.1 ± 13.4	30.8 ± 13.5	**35.3** ± 14.5**	**29.8** ± **13.6**	33.2 ± 14.7	29.9 ± 13.1

Using two-tailed paired samples t-test. The results are shown as mean ± standard deviation, with volume values adjusted to the total striatal mask volume. The compartmental volumes and connectivity indices used are contralateral to the side shown. Volumes are measured in cm^3^, iSDs in %, and MCSs are measured as a connectivity score.

*MCS* mean connectivity score, *iSD* index of streamline density, *SBR* specific binding ratio.

*<0.05; **<0.01

*p* < 0.05 values are highlighted in bold.

## Discussion

Our results show changes in striatal matrix-like and striosome-like related connectivity that correlate with the level of nigrostriatal denervation in treatment-naïve PD patients. Matrix-like connectivity changes were found to be more pronounced; matrix-like compartment volume was higher in cases where putaminal SBR was lower, consistent with nigrostriatal denervation. These changes seemed to be caused by higher mean connectivity scores and higher percentages of voxels connecting to the matrix-favoring targets. The changes in striosome-related connectivity were significant only when comparing hemispheres with higher vs. lower nigrostriatal denervation, and changed in patterns opposite those of matrix-related changes; the striosome-like compartmental volume and the number of voxels connecting to striosome-favoring targets being lower on the side with more pronounced nigrostriatal denervation. Clinical indices followed a similar trend, with lateralized MDS-UPDRS III scores being associated with a higher matrix-like volume on the contralateral side and vice versa for both the striosome- and matrix-like volumes. Out of the motor subscores tested, the rigidity subscore showed the most consistent associations with compartmental volumes and connectivity indices. These changes were not driven by differences in cortical thickness or mask volume of tractography target structures nor by changes in total volume of striatal masks, putamina or caudate.

As previously stated, the applied method assigns to striatal voxels a connectivity profile consistent with striosomes and matrix but does not directly measure their volume in the striatum. The only published histopathological study did not find changes in the volume of striosomes and matrix in PD^27^, however the study had notable limitations. In contrast to our research, it included only 3 patients with PD and 8 control subjects, evaluated immunoreactivity at single planes rather than providing a volumetric analysis across the whole striatum, and did not attempt to correlate possible compartmental changes with the level of nigrostriatal denervation. Nonetheless, the observed changes in compartmental volume are more likely caused by alteration of connectivity than by actual degeneration of striosomal and proliferation of matrix striatal compartments. It is also not known whether nigrostriatal denervation in PD and iRBD (when present) preferentially alters one striatal compartment. Previous studies using MPTP-treated animals or mice with *weaver* mutation yielded conflicting results, with some reporting preferential loss of dopamine terminals in striosomes^29,33^ and others in matrix^30,34^.

Several studies with different approaches examined possible structural white matter alterations in PD and some found direct or indirect signs of increased connectivity in early PD and its prodromal stages^35^. These include, among others, connections involving cortical and subcortical regions that are specific for PD connectome^36^ and increased fractional and axial anisotropy in the supplementary motor area-putamen tracts^37^. However, even in early stage PD, the findings of increased connectivity are not consistent^38^. It has also been demonstrated that different PD motor subtypes show distinct patterns of connectivity changes^39–41^.

Interpretation of probabilistic tractography results is also notoriously complex, as a higher connectivity score does not equal a “stronger” white matter tract. Connectivity scores are dependent on several factors other than the number of axons in a pathway, including, but not limited to, the pathway’s length, curvature, branching, and a proximity of other white matter tracts with different directions. Moreover, tractography in general does not differentiate the direction of tracts, meaning the changes in connectivity may both be afferent and efferent in nature^42^. Our study, however, almost exclusively uses cortical target regions. As direct pathways between the cortex and the striatum are unidirectional, with cortex projecting to the striatum^43^, it is reasonable to assume we are primarily assessing striatal afferents. The indirect striato-cortical connections are likely to contribute comparatively less to the overall connectivity score, as indirect pathways would be longer, more curved and branched, thus harder to identify by probabilistic tractography. This, in turn, reduces the variance that can be introduced when assessing regions with reciprocal connectivity. The parcellation method we used is also by itself less affected by inter-individual and inter-scan variance, as it depends on differential connectivity, not absolute connectivity indices.

The changes we found suggest a nigrostriatal denervation-dependent shift in connectivity towards striatal matrix-like voxels with less-consistent signs of decreased connectivity to striosome-like voxels. These alterations were also in part associated with clinical manifestations when comparing the sides with lower and the sides with higher lateralized MDS-UPDRS III scores and subscores on a within-subject level. The lack of significance when correlating connectivity indices with motor scores on a between-subject level could be theoretically attributed to considerable variance in connectivity among studied individuals.

There are several points at which pathological findings in PD could alter striosome and matrix connectivity. These include 1) differential loss of nigrostriatal innervation of striosomal or matrix MSNs or 2) differential loss of nigrostriatal innervation in whole striatal areas that have higher predominance of striosomes or matrix and 3) compensatory changes for dopamine loss in PD^44^ that could preferentially impact either striosomes or matrix, be it in the context of direct and indirect pathways or in the wider context of corticostriatal connections. The observed increase of matrix-like compartmental volume and matrix-associated connectivity to matrix-favoring regions (which include primary and supplementary motor cortex) in early-stage PD could be explained by preferential nigrostriatal denervation of the putamen which is considered the “motor” part of the striatum^45^, thus initiating a compensatory increase in connectivity. The interplay of preferential denervation-compensatory increase in connectivity could also be more nuanced based on topographical organization of cortical inputs to striatum, which are less clean cut than a simple division of putamen/striatum^45^. On the other hand, the decrease in striosome-like compartmental volume and striosome-associated connectivity indices could, for example, be explained by preferential degeneration of ventrolateral SNc in PD, as ventral tiers of SNc predominantly connect to striosomes. Further studies will be needed to elucidate the precise spatial organization of changes in striosome- and matrix-like compartments in PD and to ascertain which compartment-favoring regions contribute to these changes the most. We can exclude the effect of dopaminergic medication on our findings as our PD and iRBD participants did not receive dopamine supplementation. In this regard, it will be interesting to examine the effect of dopaminergic medication on striatal compartmental connectivity and its relation to drug-induced dyskinesias in future studies. Another potential avenue for further investigation was also introduced in a recent study that employed probabilistic tractography to segment the striatum of PD and iRBD patients, followed by a subsequent analysis of the resulting compartments using QSM^46^.

The major limitation of our study lies in the significant inter-individual variation in striatal matrix and striosomal values, which prevents applying this method at an individual level. However, future studies using improved DTI techniques might address this issue. Despite these limitations, the findings from this proof-of-concept study enhance our comprehension of PD pathophysiology.

In the previous two studies^32,47^ using this method, the bias threshold used for compartment identification was 87%. However, in this study, at times a more strict threshold of 95% yielded marginally better results in differentiating our subject groups. Thus, it should be considered that future studies use a threshold of 95% for striosome- and matrix-like voxel identification.

In conclusion, we showed a shift in striatal connectivity associated with either matrix-like or striosome-like voxels that correlated with nigrostriatal denervation and less consistently with clinical severity. An increased volume of the striatum with a connectivity profile of matrix and, less consistently, a decreased volume of the striatum with a connectivity profile specific for striosomes was associated with the degree of nigrostriatal degeneration. These results contribute to our understanding of the pathophysiology of Parkinson’s disease and open a new avenue in utilizing tractography to uncover novel insights into the disease’s progression and manifestation.

## Methods

### Participants

Our subject population consisted of de novo diagnosed treatment-naïve patients with Parkinson’s disease, isolated REM behavioral disorder patients and healthy controls recruited at the Department of Neurology, First Faculty of Medicine, Charles University and General University Hospital in Prague during 2015–2021. All PD patients were part of the BIO-PD cohort described previously^48^ and were diagnosed according to the Movement Disorders Society (MDS) clinical diagnostic criteria^2^. All iRBD patients were diagnosed in accordance with the International Classification of Sleep Disorders, third edition (ICSD-3) using video-polysomnography^49^. Patients with secondary RBD, such as related to medication usage, narcolepsy, focal brainstem lesions, and with clinically manifest parkinsonism or dementia, were excluded. The control subjects were recruited from the general community *via* advertisements. Eligibility criteria included the absence of significant neurological disorders, active oncologic illness, and abuse of psychoactive substances. RBD was excluded in all control subjects by history and video-polysomnography. All study participants were examined according to a complex protocol including structured interview, neurological examination, MDS-sponsored Revision of the Unified Parkinson’s Disease Rating Scale (MDS-UPDRS), and Montreal Cognitive Assessment (MoCA). The study was approved by the local Ethics Committee and participants signed informed consent before entering the study, in accordance with the Helsinki Declaration.

### Imaging acquisition protocol

MRI examination was performed on a 3T scanner (Siemens Skyra 3T, Siemens Healthcare, Erlangen, Germany) with a 32-channel head coil. The protocol included axial 3D T1-weighted Magnetization Prepared Rapid Gradient Echo (MPRAGE, repetition time (TR) 2,200 ms; echo time (TE) 2.4 ms; inversion time (TI) 900 ms; flip angle (FA) 8°; field of view (FOV) 230 × 197 × 176 mm; voxel resolution 1 × 1 × 1 mm^3^) and a diffusion tensor MRI with repetition time (TR) = 10.5 s; echo time (TE) = 93 ms; total 72 slices with voxel resolution of 2 mm isotropic; 30 noncolinear directions with b-value of 1000 s/m^2^ and one b = 0 s/m^2^ image.

In PD and iRBD patients, dopamine transporter single-photon emission computed tomography (DAT-SPECT) was performed using the [123I]-2-b-carbomethoxy-3b-(4-iodophenyl)-N-(3-fluoropropyl) nortropane ([123I]FP-CIT, DaTscan®, GE Healthcare, Little Chalfont, Buckinghamshire, UK) tracer according to European Association of Nuclear Medicine (EANM) procedure guidelines (Darcourt et al. 2010); the detailed protocol is described elsewhere^50^. Automated semi-quantitative analysis was performed using the DaTQUANT v. 2.0 software (GE Healthcare, USA) and specific binding ratios (SBR) in both putamina relative to background binding were calculated. DAT-SPECT was performed within one month from MRI. All PD patients were scanned before the introduction of dopaminergic therapy.

### Segmenting the striatum

Our processing protocol was based on the method established in the paper of Waugh et al.^32^. In short, we employed striosome- and matrix-favoring brain areas as target regions for quantitative probabilistic diffusion tractography (reviewed by Waugh at al.). These target regions served to segment the striatum into voxels with striosome-like or matrix-like connectivity profiles. We chose 5 matrix-favoring (primary motor cortex, supplementary motor area, superior parietal lobule, primary somatosensory cortex, and *pars opercularis* of inferior frontal gyrus) and 5 striosome-favoring (posterior part of orbitofrontal gyrus, anterior insular cortex, basolateral amygdala, basal operculum, and posterior temporal fusiform cortex) regions as probabilistic tractography targets. We planned to study the subcortical gray matter’s connectivity to striosome-like and matrix-like compartments in future studies, as the subcortical gray matter is intrinsically connected to the pathophysiology of PD. Therefore, to avoid potential issues of circularity in the future, we preferred cortical regions over subcortical nuclei as striosome- and matrix-favoring target structures. This approach follows the methodology used in a recent study by Funk et al.^47^. The regions of interest (ROIs) we used, including the striatal and striosome- and matrix-favoring target masks, were kindly provided by Waugh et al. As per their initial study, the striatal ROIs did not include the nucleus accumbens, as it does not follow the same striosome/matrix architecture of the rest of the striatum^51^, and the tail of the caudate nucleus, due to its relatively small cross-sectional area and resulting difficulties in automatic registration to the 2 mm isotropic resolution^32,47^. Both striatal and compartment-favoring masks are available as a supplementary material in the paper by Funk et al.^47^.

For image preprocessing and tractography steps we used FSL and created an automatic pipeline in Snakemake software, with the most resource-intensive computing being done on the MetaCentrum distributed computing infrastructure^52–55^. The whole analysis was done in each subject’s native diffusion space and the DWI data were corrected for distortion, eddy currents and movement artefacts using FSL’s *topup* and *eddy* tools. Resulting images were further processed using FSL’s *bedpostX_gpu* to model crossing fibers within each voxel of the brain – a necessary step for subsequent probabilistic tractography. To transform all region of interest masks from MNI152 space to each subject’s native diffusion space, T1 image of each participant was transformed into MNI152 space using FSL’s *flirt* and *fnirt*; the original T1 image was also coregistered to its corresponding diffusion image using *flirt*. All the resulting transformations were visually checked for errors. Subsequently, we used the *convertwarp* function to create a singular transformation from MNI152 space to the subject’s native diffusion space. As the transformation did not perfectly account for ventricular atrophy, we used FSL’s FAST segmentation tool to segment cerebrospinal fluid compartment and subtract it from the striatal mask in the subject’s native diffusion space. This eliminated possible overreach of striatal mask into the lateral ventricles.

**Fig. 4:**
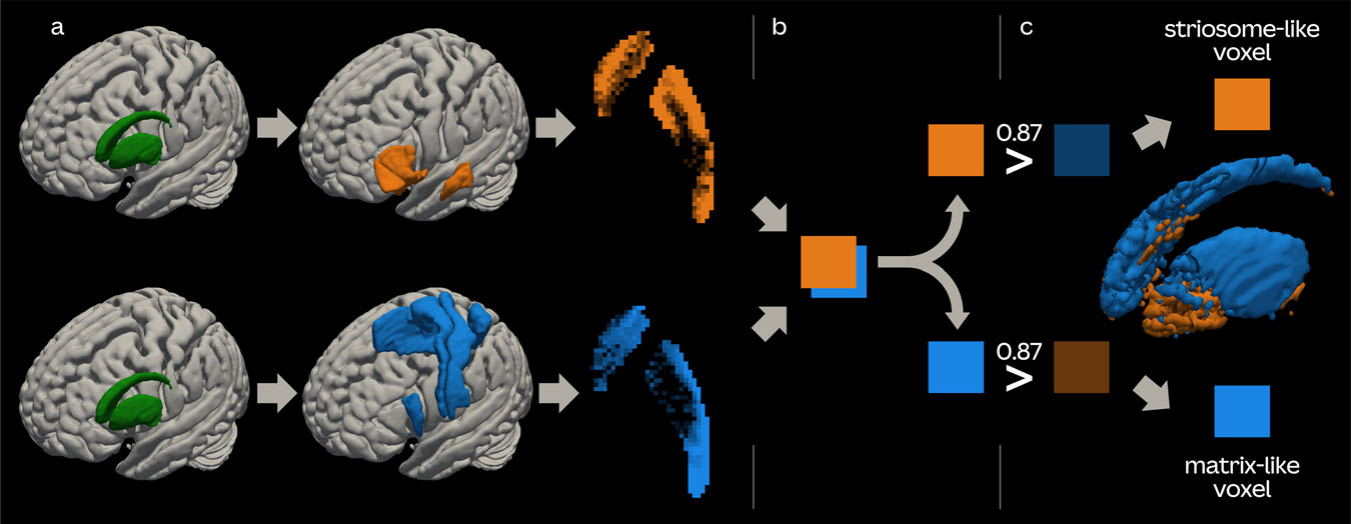
The algorithm of striosome- and matrix-like voxel identification. **a** Probabilistic tractography is initiated using the striatum as a seed and striosome- and matrix-favoring brain regions as targets. The resulting two images contain voxel values which represent the tractographic scores. **b** These images are then processed using FSL’s *proj_thresh* tool, which sums the voxel values of both images and generates a summed image and two new striosome- and matrix-associated images. The value of a voxel in the two newly generated images represents a fraction of the respective compartment’s tractographic score in the summed image. **c** If in either of these images a voxel value exceeds 0.87, the voxel is identified as striosome- or matrix-like, respectively.

We performed classification targets probabilistic tractography using FSL’s *probtrackX*. Each ipsilateral striatum was used as a seed and the composite matrix- and striosome-favoring masks were used as targets. A midline exclusion mask was used to ensure that all resulting connections were ipsilateral. We included a distance correction option to reduce the impact of path length on streamline completion. The other settings remained as default: curvature threshold = 0.2; steplength = 0.5 mm; number of samples = 5000; number of steps per sample = 2000. Subsequently, we used the *proj_thresh* tool on the *probtrackX*’s striatal tractographic score images to determine each voxel’s connectivity bias towards either matrix- or striosome-favoring targets. Voxels were labeled as striosome-like or matrix-like if they had a connection predominance to the specified target of at least 0.87 (a fixed threshold we empirically found in prior studies yielding good representation of voxels from both compartments). In addition to the empirical threshold of 0.87, we also tested a more strict threshold of 0.95 to examine the robustness of the method. The volumes of striosome-like and matrix-like compartments were calculated as sum of all voxels with connection predominance to striosome- or matrix-favoring targets, respectively (Fig. 4).

For each of the two compartments we also calculated two additional connectivity metrics:

1) A mean connectivity score (MCS) of the whole striatal mask, calculated as a sum of all striatal voxels’ connectivity scores to a target mask divided by the number voxels in the striatal mask.

2) An index of streamline density (iSD), calculated as a percentage of voxels in the whole striatal mask with at least one streamline seeded to the target mask.

To rule out the possible effect of cortical thinning on connectivity, we calculated the mean cortical thickness of the two composite ROIs. We used *freesurfer*’s *recon-all* tool to calculate the cortical thickness of the whole brain, and extracted the mean cortical thickness of striosome- and matrix-favoring composite target masks using *freesurfer*’s *mri_vol2surf*, *mri_cor2label* and *mris_anatomical_stats* tools^56–58^. To examine other automatic striatal segmentation approaches that could differentiate our subject groups, we used *recon-all*’s automatic segmentation output to acquire volumes of putamen and caudate and calculated their volume in both hemispheres combined. To acquire the value of total intracranial volume (TIV) we have used the automatic segmentation pipeline of the Computational Anatomy Toolbox CAT12^59^. We did not use *recon-all*’s estimated total intracranial volume, as it is prone to bias due to brain atrophy^60^.

### Statistics

All statistical analyses were performed using IBM SPSS for Windows, Version 26. For demographic and clinical characteristics, we utilized one-way ANOVA to analyze the inter-group differences in age, and the Chi-squared test to compare the sex distribution among groups. We also used one-way ANCOVA with age and sex as covariates to measure the differences in MoCA, MDS-UPDRS III score, and the mean cortical thickness of composite target masks; the same covariates with the addition of TIV were used to compare the total volumes of striatal seed mask, striosome- and matrix-favoring target masks, putamina, and caudates between groups.

Volumes of striosome- and matrix-like compartments were adjusted for total striatal mask volume (TSMV) using the regression coefficient β and TSMV_mean_ from control subjects as follows^61^:

1 $${{\rm{Volume}}}_{{\rm{adjusted}}\,i}={{\rm{Volume}}}_{{\rm{raw}}\,i}-{\rm{\beta }}\,\left({{\rm{TSMV}}}_{{\rm{raw}\,i}}-{{\rm{TSMV}}}_{{\rm{mean}}}\right)$$

Using one-way ANCOVA with age and sex as covariates, we assessed the inter-group differences in adjusted total striosome- and matrix-like compartmental volume as well as in MCS and iSD of PD, iRBD, and control subjects. The measure of effect size was expressed as partial eta squared (partial η^2^). All post-hoc multiple comparisons were performed using the least significant difference.

To describe the effects of nigrostriatal denervation on the connectivity, we pooled all our subjects with DAT-SPECT (PD *N* = 72 and iRBD *N* = 53). We used the mean putaminal SBR from both hemispheres, which is inversely proportional to nigrostriatal denervation. Using partial correlation with age and sex as covariates of no interest we analyzed the relation of mean putaminal SBR to total compartmental volumes, and mean MCSs and iSDs. To assess the intra-subject effect of nigrostriatal denervation on compartmental volumes, MCSs and iSD we used a paired Student’s t-test to compare the side with higher and the side with lower putaminal SBR on DAT-SPECT.

To determine the correlation between motor symptoms and connectivity in PD patients (*N* = 72) we used MDS-UPDRS III score and its tremor (items 15–18), bradykinesia (items 2, 4–9 and 14), rigidity (item 3), and axial (items 1 and 9–13) subscores^62^. We used partial correlation with age and sex as covariates of no interest to analyze the relationship between compartmental volumes, iSDs and MCSs with MDS-UPDRS III scores and subscores. Subsequently, we used paired Student’s t-test to compare the side with higher and the side with lower lateralized MDS-UPDRS III score, excluding items that are not lateralized (items 1, 2, 3a, 9–14, 17e, and 18).

Since the iRBD group was significantly older than the PD and control groups, we performed sensitivity analyses in age-matched subgroups to exclude potential age-related bias; age matching was performed using the SPSS’ case control matching utility, with randomized case order when drawing matches and a match tolerance value of 5.

### Supplementary information

Supplemental material

## Data Availability

The datasets used and analyzed during the current study are available from the corresponding author on request.
