# Supplementary material for: Analysis of striatal connectivity corresponding to striosomes and matrix in de novo Parkinson’s disease and isolated REM behavior disorder
Source: NPJ Parkinsons Dis. 2024 Jun 25;10:124. doi: 10.1038/s41531-024-00736-9 (PMC11199557; doi:10.1038/s41531-024-00736-9)
Supplement: Supplementary file 1 — Supplemental material [file 41531_2024_736_MOESM1_ESM.docx]

| Calculations using the 95% threshold for striosome- and matrix-like voxels | | | | |  |  |
| --- | --- | --- | --- | --- | --- | --- |
|  |  |  |  |  |  |  |
| Supplementary Table 1: Comparison of compartmental volume in PD, iRBD and controls using the 95% threshold | | | | | | |
|  | Controls (n=45) | iRBD (n=56) | PD (n=72) | Inter-group *p*-value | partial η^2^ | Between groups |
| Matrix-like volume [cm^3^] | 5.4±1.0 | 5.6±0.9 | 5.9±1.0 | **0.015** | 0.049 | PD>>C* |
| Striosome-like volume [cm^3^] | 2.3±0.9 | 1.9±0.7 | 2.2±1.0 | 0.179 | - | - |
| Where applicable, the results are shown as mean ± standard deviation, with volumetric values adjusted to the mean total striatal mask volume. *C* controls, > for p<0.05, >> for p≤0.01, * p=0.004 | | | | | | |

| Supplementary Table 2: Associations of nigrostriatal denervation (SBR) and compartmental volumes using the 95% threshold | | |
| --- | --- | --- |
|  | Partial correlation coefficient | p-value |
| Matrix-like volume | -0.226 | **0.012** |
| Striosome-like volume | -0.035 | 0.703 |
| Using two-tailed partial correlation, n=125. Controlling for age and sex. | | |

| Supplementary Table 3: Associations of MDS-UPDRS III and its subscores with compartmental volumes using the 95% threshold | | | | | |
| --- | --- | --- | --- | --- | --- |
|  | MDS-UPDRS III | Tremor | Rigidity | Bradykinesia | Axial |
| Matrix-like volume | **0.026 (0.171)** | 0.062 (0.143) | 0.085 (0.132) | **0.027 (0.169)** | 0.193 (0.100) |
| Striosome-like volume | 0.827 (0.010) | 0.958 (-0.004) | 0.269 (0.085) | 0.938 (-0.006) | 0.997 (0.000) |
| Using two-tailed partial correlation, n=173. Controlling for age and sex. Data are shown as p (partial correlation coefficient). Significant correlations are highlighted. | | | | | |
